# Clinical- and Cost Effectiveness of a Guided Internet-Based Intervention for Children (12–18 Years) of Parents With Mental Disorders (iCHIMPS): Study Protocol of a Multicentered Cluster-Randomized Controlled Trial

**DOI:** 10.3389/fdgth.2022.816412

**Published:** 2022-02-16

**Authors:** Patrick Dülsen, Katja Barck, Anne Daubmann, Alexandra Höller, Jan Zeidler, Reinhold Kilian, Silke Wiegand-Grefe, Harald Baumeister

**Affiliations:** ^1^Department of Clinical Psychology and Psychotherapy, Institute of Psychology and Education, Ulm University, Ulm, Germany; ^2^Department of Medical Biometry, Institute for Medical Biometry and Epidemiology, University Medical Center Hamburg-Eppendorf, Hamburg, Germany; ^3^Center for Health Economics Research Hannover (CHERH), Leibniz University Hannover, Hanover, Germany; ^4^Department of Psychiatry II, BKH Günzburg, Ulm University, Ulm, Germany; ^5^Department of Child and Adolescent Psychiatry, Psychotherapy and Psychosomatics, University Medical Centre Hamburg-Eppendorf, Hamburg, Germany

**Keywords:** e-mental-health, psychotherapy, children and adolescents, parents with mental disorders, internet- and mobile-based intervention

## Abstract

**Introduction:**

Children of parents with mental disorders have a high chance of developing a mental disorder themselves. However, this at-risk group is regularly overlooked and typically not seen by any mental health professionals. Internet- and mobile-based interventions (IMIs) can provide a means of promoting mental health for children of parents with mental disorders.

**Objective:**

The introduced study will evaluate the clinical- and cost-effectiveness of the iCHIMPS IMI in promoting mental health for children of parents with mental disorders.

**Methods:**

A two-armed multicentered cluster-randomized controlled trial (cRCT) comparing the clinical- and cost-effectiveness of the iCHIMPS IMI in the intervention group (IG) to a treatment-as-usual (TAU) control group will be conducted. Recruitment will be handled at currently 21 adult mental health clinics throughout Germany. Participating families will be randomly divided into the two groups until the final sample size of 306 participating adolescents (age 12–18) has been reached. The adolescents in the intervention group will receive access to the IMI and can take part in up to eight intervention modules. Assessment will be conducted during the recruitment (baseline), 1-month, 2-months, and 6-month post-inclusion. Primary outcome is the mental health of the participating adolescents at 6-months post-inclusion as measured by the Youth Self Report score. Secondary self-report outcomes are mental wellbeing, self-efficacy, coping strategies and negative effects as well as mental health of the adolescents as reported by their parent(s). Included moderators are sociodemographic characteristics, working alliance, social support and the mental health diagnoses of the parents. Statistical analyses will be conducted on the intention-to-treat principle as well as with additional per-protocol analyses. Additionally, the cost-effectiveness as well as qualitative data concerning the adherence, acceptance, and feasibility of the IMI will be analyzed.

**Discussion:**

The iCHIMPS cRCT examines the clinical- as well as cost-effectiveness of the iCHIMPS mental health promotion IMI for children of parents with mental disorders. This provides the opportunity to gain insights into an innovative as well as time- and location-independent form of support for this often-overlooked at-risk group. Additionally, the larger CHIMPS-NET project allows comparisons between internet-based and face-to-face interventions for a similar target group.

**Clinical Trial Registration:**

www.ClinicalTrials.gov, identifier: DRKS00025158.

## Introduction

An estimated 25% of children and adolescents throughout Germany have at least one parent with a mental disorder (1). The chances of developing any mental disorder for the children themselves have been reported to be as high as 50%, and 33% for severe mental disorders (2). Wiegand-Grefe and colleagues (3) highlighted a 3–7 times higher risk of showing symptoms of any mental disorder when compared to the general population. Children of parents with a mental disorder, however, are typically not seen by mental health professionals (4, 5). One reason for the limited health care coverage might result from the lack of mental health promotion programs specifically tailored to the needs and demands of this at-risk group (6).

Reasons for the vulnerability to mental disorders of children and adolescents with parents with mental disorders are reflected by the complex and heterogenous psychosocial, bio-genetic as well as medical situation of every single family member (5, 7). For example, the parent with a mental disorder can have diagnoses ranging across the whole spectrum of mental disorders. Their partner can either be healthy, on the threshold of developing a mental disorder, mentally ill themselves or mentally ill but not yet diagnosed. On the other hand, the children can be either resilient and not symptomatic, they can be on the threshold or they can already show clinically relevant mental symptoms themselves (7). The huge amount of possible multi-way interactions of these risk- and resilience-factors demonstrate the challenges as well as opportunities posed to the care of such children.

Treatment and prevention approaches for mental disorders based on psychotherapeutic techniques are effective interventions for adults as well as children and adolescents (8–12). Preventive interventions focusing on families with at least one parent with a mental disorder seem to be capable to effectively reduce the risk of developing a mental disorder for the children within these families by up to 40% (13). However, most of the available interventions target the parents and are therefore not readily suitable for the children themselves, leaving almost no specific interventions for children (6). An additional reason for children of parents with a mental disorder not receiving the professional help they might need is the often existing mental health care gap (14, 15) between people in need of care and available programs. It has further been argued that this gap as well as the quality and specificity of available psychotherapeutic interventions can be even more limited for children and adolescents (16). A potential approach to decrease this gap might be given in the form of Internet- and mobile based self-help interventions (IMIs) (17, 18). Especially the younger generation with their generally high digital affinity might be more open to use these kinds of mental health promotion interventions.

IMIs can be characterized by their mode of delivery e.g., via webpages or mobile apps and often consist of persuasive features such as videos, audios, and chat functions (19). Furthermore, IMIs are usually grounded on an evidence-based theoretical background, most often cognitive behavioral therapy, but also other approaches such as psychodynamic therapy are available. Additionally, the amount of human support provided by IMIs may vary (17, 20). The benefits of internet-based interventions, such as their time- and location-independent form of delivery as well as the personnel-efficient procedure, can increase the amount of mental health promotion programs that are provided to the population (17). There is already substantial evidence for the effectiveness of IMIs for children and adolescents who suffer from mental disorders [e.g., (18, 21, 22)]. Evidence of the effectiveness of IMIs for children of parents with a mental disorder is scarce but promising (23–25). Reupert and colleagues (24) reported a reduction in depression and stress scores while another group of researchers stated an improvement in coping and mastery mechanisms after participating in their IMI (25).

Meeting the aforementioned complex needs of families with at least one parent with a mental disorder is the main goal of the joint multicenter research project CHIMPS-NET (CHIldren of Mentally ill ParentS - NETwork). The research project investigates the possibilities of breaking through the circle of transgenerational transmission of mental disorders. For this purpose, three face-to-face prevention and treatment interventions based on the CHIMPS-program (26) are already being evaluated. The present study will evaluate the clinical- as well as the cost-effectiveness of iCHIMPS, a mental health promotion IMI. The intervention was specifically developed for adolescents with parents that have a mental disorder and is generically designed to be used by adolescents who either already show symptoms of mental disorders themselves or not. The overarching goal of the internet-based intervention is the promotion of mental health and the improvement of self-management abilities among adolescents (age 12–18 years). In detail the present trial will examine the following:

1 The clinical-effectiveness of iCHIMPS compared to TAU (treatment-as-usual) regarding mental health at 6-months follow-up
2 The clinical-effectiveness of iCHIMPS compared to TAU (treatment-as-usual) regarding the secondary outcomes (mental-wellbeing, further mental health outcomes, self-efficacy and coping strategies)
3 The cost-effectiveness of providing iCHIMPS in addition to TAUvs. TAU alone
4 Quantitative and qualitative information about the adherence and acceptance as well as potential barriers and facilitators of iCHIMPS
5 Moderators and mediators of intervention success as well as potential adverse events.

## Methods

### Study Design

A two-armed multicentered cluster-randomized controlled trial (cRCT) comparing the clinical- and cost-effectiveness of the iCHIMPS IMI in the intervention group (IG) to a treatment-as-usual (TAU) control group will be conducted.

The study has been approved by the Ethics Committee of Ulm University, Germany (189/20-FSt/bal.) and has been registered in the German clinical trial register (DRKS00025158). Results will be reported according to the Consolidated Standards of Reporting Trials (CONSORT) Statement 2010 and the extensions for reporting cluster randomized trials and trials on psychological interventions (27, 28). The cost-effectiveness analyses will be reported in accordance with the Consolidated Health Economic Evaluation Reporting Standards Statement (29), as well as with the guidelines of the International Society for Pharmacoeconomics and Outcomes Research (30). This trial protocol was created according to SPIRIT guidelines (31).

### Participants

#### Cluster Definition

The study population are families, which are defined as (a) having at least one parent with a mental disorder according to the ICD-10 [mental disorder is defined by a mental disorder diagnosis made by a licensed health care professional (i.e., physician or psychotherapist) within the last 6 months] as well as (b) at least one child between the age of 12 years and 0 months to 18 years and 11 months.

Additionally, all participating family members (at least the parent with the mental disorder as well as one child) need to (c) have access to a computer, laptop or smartphone as well as to the internet, and have sufficient proficiency in German.

Whole families as a cluster and not the separate family members will be randomized, due to the possibility that siblings from the same family could be randomized into different study groups. Siblings in two different study groups would potentially increase the risk of group contamination and thereby compromise the validity of the study results.

The trial focuses on the children and therefore the parents do not necessarily have to participate in the study, except for giving informed consent and providing information about their mental disorder at the baseline assessment, and the children do not necessarily have to live with their parent(s) or the parent(s) do not necessarily have to have custody of the participating children. For a better readability, the participating children will be referred to as participants hereafter, while their parent(s) and if applicable legal guardian(s) will be called parents.

#### Inclusion and Exclusion Criteria of Participating Family Members

##### Participants

All participants need to (a) have signed the informed consent [if the participating adolescent is below 16 years of age, signed informed consent by the parent(s) or, if different custody arrangements apply then the legal guardian(s)] and (b) have successfully completed the baseline assessment.

Exclusion criteria are (a) acute suicidal tendencies, (b) acute substance use disorder (ICD F1X.2 except nicotine dependence F17.2) or (c) acute psychotic symptoms exhibited by the participating adolescent as assessed by clinical professionals at the participating clinics at baseline.

##### Parent(s) and/or Legal Guardian(s)

At least one parent with a mental disorder needs to have (a) signed the informed consent and (b) taken part in the baseline assessment.

Further participating family members need to have (a) signed the informed consent.

##### Further Participation in the Assessments

The parent(s) and if applicable the legal guardian(s) will be encouraged to take part in the whole assessment procedure, however, their non-participation, apart from the baseline assessment, will not be considered an exclusion criterion.

#### Sample Size and Power Calculations

The sample size calculation was conducted with the Power Analysis and Sample Size Software (PASS 15) and is approximated based on the “Tests for Two Means in a Cluster-Randomized Design” module. As clinically significant mental health effect a moderate effect size of 0.5 (Cohens *d*) in the primary outcome (Youth Self Report score; YSR 11-18R; 32) between the two groups (iCHIMPS vs. TAU) at 6-months post inclusion has been assumed. Calculating with a power of 80%, a significance level of 0.5% (two-sided hypothesis), an inter-cluster correlation coefficient of 5%, and a family size of 1.3 children between the age of 12 years and 0 months and 18 years and 11 months per family, 65 participants in 50 families per group (130 participants in 100 families in total) are needed. Considering a drop-out rate for online-interventions of 50–60%, our trial will aim at 306 participants in 234 families (153 participants in 117 families per group) as has been estimated in the original grant application.

### Recruitment

The recruitment has started on May 1^st^ 2021 and will be handled at 21 adult mental health clinics across Germany. Adult mental health clinics have been chosen as recruitment sites due to the fact that children of parents with a mental disorder are regularly overlooked for prevention or treatment offers and are therefore often not or not yet seen by any mental health professional, hence not reachable via the standard mental health care system for children and adolescents. Study employees at the participating adult mental health clinics will additionally recruit potential families from other mental health care locations or through advertising.

### Randomization

Participating family-clusters will be allocated in a 1:1 ratio to either iCHIMPS or TAU group according to a central randomization list based on block wise randomization with a variable block length and without stratification. The randomization list was generated by the Institute of Medical Biometry and Epidemiology of the University Medical Center Hamburg-Eppendorf using the statistical software R version 3.6.3.

#### Blinding and Masking

The randomization as well as the final statistical analysis will be conducted by scientists at Ulm University not otherwise involved in the present trial. The study team, which designed the intervention and assessment, will not be blinded toward participant features or group memberships. Reasons for this approach are feasibility limits and the offered support via email given to the participants. However, since the main risk is posed by unblinded randomization and statistical analysis, we are confident the selected approach is well suited to deliver unbiased results.

### Procedure

**Figure 1:**
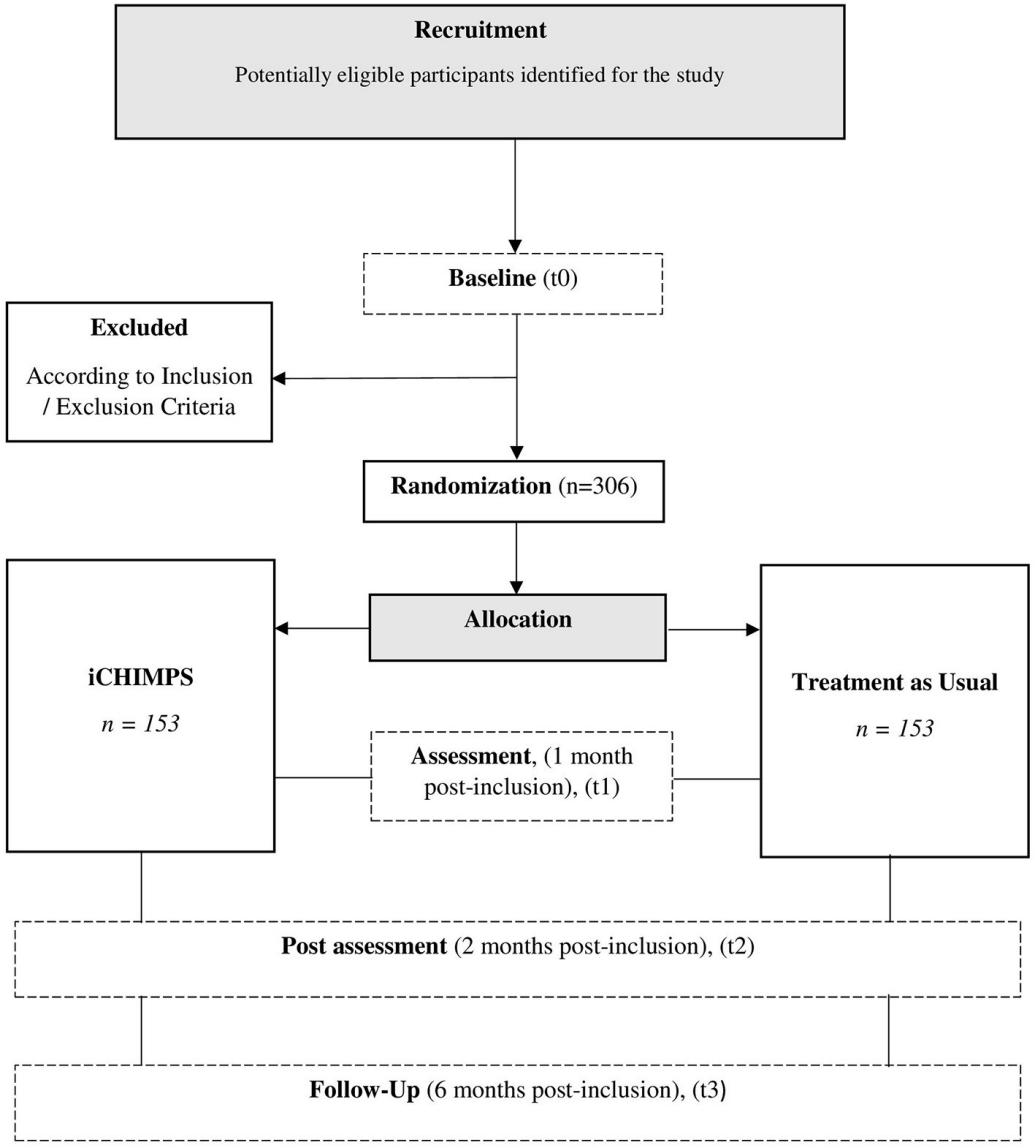
Flow chart.

At each participating adultmental health clinic an employee, who is briefed about the study, recruits potential families via mental health care locations or advertisements (see Figure 1 for a flow chart representation).

If the potential family meets the inclusion criteria for families and all necessary parties have signed the informed consent as well as the contact forms, a clinical assessment interview will take place, either at the clinics or via phone. The inclusion and exclusion criteria will be written down and transferred to the study team at Ulm University together with the other forms. This marks the end of the recruitment procedure at the adult mental health clinics.

After the recruitment at the clinics is finalized and the inclusion criteria are fulfilled, the participants and possibly their parents and/or legal guardian(s) will receive a personalized link for the online assessment. After the successful completion of the online baseline assessment (t0) by at least the participant and the parent with the mental disorder, the family is eligible to be included into the trial and will be randomized according to the randomization list by a scientist at Ulm University.

Depending on the group the family was randomized to, an email will contain the explanation of the procedure of the internet-based intervention and the following online assessments (iCHIMPS) or the explanation of why they will not receive access to the internet-based intervention and the procedure of the following online assessments (TAU).

If the participant was assigned to iCHIMPS, he or she is able to log into the internet-based intervention at https://patient.esano-trainings.de and complete the eight consecutive modules of the internet-based intervention at her or his own pace.

All participants (ICHIMPS and TAU) and possibly their parent(s) and/or legal guardian(s) will receive emails with an invitation to the online assessments for t1 (one-month post-inclusion), for t2 (two-months post-inclusion) and for t3 (six-months post-inclusion).

### Intervention

The internet-based intervention iCHIMPS was developed by the study team of the Department of Clinical Psychology and Psychotherapy at Ulm University and builds onto previously devised internet-based interventions of the department (33) as well as on the CHIMPS-program (26). It is a generically designed intervention suitable for children and adolescents, with a parent having a mental disorder, who either already show symptoms of mental disorders themselves or not. The intervention was designed in such a way that it can be carried out by the children and adolescents without any help by an adult, meeting the needs of the potentially complex and strained family situations. The goals of iCHIMPS are to enable children of parents with a mental disorder to be able to better deal with their difficult life situation, as well as to increase their mental health, quality of life and self-management abilities.

iCHIMPS is designed as a series of eight consecutive modules. The content is delivered in text, picture, video as well as audio format and can be partly customized to the individual interests of the user. iCHIMPS is based on four main principles: (1) The intervention is internet and mobile based and can be accessed from any computer, laptop or smartphone, thereby being optimized for devices with larger screens and keyboards. (2) To increase the adherence of the users the design follows persuasive principles (19) and motivational messages as well as sending reminders regularly. Furthermore, after each session a fixed date will be arranged on when the next module will be started. (3) It is based on an evidence based theoretical background combining cognitive behavioral and psychodynamic therapy approaches (26, 33). (4) To facilitate the transfer of what the participants have learned during the intervention into their daily lives, interactive home tasks and helpful suggestions in terms of take-home-messages will be provided. Some of the home-tasks can be done together with other family members.

The main topics of the intervention are the engagement with the users own life situation, their own challenges, resources and strengths as well as the engagement about the mental disorder of their parent(s). Further covered topics include health, communicating about difficult topics, strengthening familial and social relationships, stress management, emotion regulation and establishing healthy boundaries to increase autonomy (an overview of the modules is provided in Table 1). The techniques used are based on cognitive behavioral therapy, such as cognitive restructuring, on mindfulness-based therapy as well as psychodynamic therapy, such as clarifying their own life situation and careful reflection about dysfunctional communication or relationship patterns.

**Table 1:** Overview of intervention modules.

**Modules**		**Content**
1	What can you expect?	Introducing the team, literature and guiding principles; Familiarising with the iCHIMPS process, overview of the modules and E-Coaching
2	The first step – get to know yourself and your challenges.	Strengths and worries of the participants; Identifying one's values, goals and resources; Challenges in everyday life with a mentally ill parent
3	Knowledge means strength – find out more about your parent's mental illness.	Psychoeducation; Family coping with illness
4	Talk about it – stay in contact and learn to talk about difficult subjects.	Relevance of communication, rules for successful communication, talking about your parent's mental illness with different groups of people
5	Find inner peace – learn how to cope with stress.	Stress management; Possibilities for coping with stress
6	The world of emotions.	Explaining Emotions; Coping with negative Feelings, promoting pleasant feelings
7	You, yourself and your rough edges – learn how to set limits.	Setting limits; Strengthen your self-esteem
8	Last stop! – What is next?	Summary of Modules 2–7; Recognition of warning signs and prevention

Additionally, the internet-based intervention can be complemented by a varying amount of human support. E-Coaches can provide guidance through individualized feedback on each section and answer potential questions in an asynchronous fashion. E-Coaches in the present study are children and adolescents' psychotherapists (in training) who undergo a specialized workshop and regular supervision.

### Intervention Platform

iCHIMPS was designed and will be made accessible on the e-health platform eSano, an open source platform developed by the Department of Clinical Psychology and Psychotherapy and the Institute of Databases and Information Systems at Ulm University (34). The platform consists of three parts: (1) the content management system, through which the intervention can be designed; (2) the E-coach platform, through which the case management, i.e., process monitoring, guidance and feedback on individual modules can be provided; and (3) the patient application (web-app) through which the participants can access the intervention. All three parts of the platform are located on secured servers and regulated by the necessary safety rules and network security devices. eSano was developed according to the requirements of the German Medial Devices Act and the Medical Device Regulation (MDR), thereby, considering the IEC 62304 (safety class B), the GAMP5 (category 4), the General Principles of Software Validation of the FDA as well as the Pharmaceutical Inspection Cooperation Scheme (PIC/S) 11-3.

### Assessments

The Assessment will be conducted with the participants and at least partly with the parent(s) and/or legal guardian(s) as online self-report questionnaires (via the platform Lime Survey), as in-person or telephone-based interviews, and by the collection of claims data through the participating health insurance companies. An overview of all assessment materials for t0 (baseline assessment), t1 (inter-session assessment 1-month post-inclusion), t2 (2-months post-inclusion) as well as t3 (6-months post-inclusion) is provided in Table 2 and will be individually introduced below.

**Table 2:** Overview assessment.

**Instrument**	**Variable**	**Time of measurement**			
		**t0**	**t1**	**t2**	**t3**
**Assessment adolescents**					
YSR 11-18R	Mental health	x	x	x	x
WHO-5	Mental wellbeing	x	x	x	x
KIDDI-SADS-PL	Mental health (interview)	x			
SES	Self-efficacy	x	x	x	x
Brief-COPE	Coping strategies	x	x	x	x
SD	Sociodemographic characteristics	x			
WAI-I	Working alliance	x	x	x	x
OSSQ	Social support	x	x	x	x
EQ-5D-Y	Health related life quality	x			x
CAMSHRI-DE	Utilization of the health care system	x			x
QO	Qualitative outcomes				x
NEQ	Negative effects		x	x	x
**Assessment parent(s) and/or legal guardians**					
CBCL 6-18R	Proxy mental health of the adolescent	x		x	x
M.I.N.I.	Mental health (interview)	x			
OSSQ	Proxy social support of the adolescent	x		x	x
SD	Sociodemographic characteristics	x			

*t0, Baseline; t1, 1 month; t2, 2 months; t3, 6 months; YSR 11-18R, Youth Self Report; WHO-5, World Health Organization-5; KIDDI-SADS-PL, Kiddie Schedule for Affective Disorders and Schizophrenia Present and Lifetime Version; SES, Self-Efficacy Scale, Brief-COPE, Brief Coping Orientation to Problems Experienced Inventory; SD, Sociodemographic characteristics; WAI-I, Working Alliance Inventory for Guided Internet Interventions; OSSQ, Oslo Social Support Scale; EQ-5D-Y, EuroQol-5-Dimensions-Youth; CAMSHRI-DE, Children and Adolescent Mental Health Services Receipt Inventory; QO, Qualitative outcomes; NEQ, Negative Effects Questionnaire; CBCL 6-18R, Children Behaviour Check List; M.I.N.I., Mini-International Neuropsychiatric Interview*.

#### Primary Outcome

The primary outcome is mental health of the participant at 6-months post-inclusion (t3) as measured by the Youth Self Report score (YSR 11-18R; 32). All 112 items will be combined to calculate a total problems score. A t-value above 63 marks the cut-off to a clinically relevant score. Internal consistency of the full scale measured by Cronbach's α is ≥0.93 (32).

#### Secondary Outcomes Participants

Mental health of the participant as measured by the Youth Self Report score (YSR 11-18R; 32). The 112 items can be divided into eight problem subscales (e.g., social problems or aggressive behaviors) and 2 second-level scales (internalizing and externalizing problems). All ten subscale scores will be calculated separately. For the eight problem subscales a t-value above 70 marks the cut-off to a clinically relevant score, while for the internalizing and externalizing problems scales a t value above 63 is considered clinically relevant. Internal consistency ranges for the subscales from α = 0.59–0.84 in a community sample and from α = 0.62–0.90 in a clinical sample (32).

Mental wellbeing of the participants will be measured with the World Health Organization-5 [WHO-5; (35)]. Internal consistency of this very economic scale with a processing time of under 1 min on average, assessed by Cronbach's α is 0.92 (36).

Mental health of the participant as assessed by the German version (37) of the semi-structured clinical interview Kiddie Schedule for Affective Disorders and Schizophrenia Present and Lifetime Version [Kiddie-SADS-PL; (38)] designed to be used by trained interviewers to assess mental disorders according to DSM-IV and ICD-10 diagnostic criteria. For the interview fair to excellent interrater reliabilities for the different scales and for different languages have been reported (39, 40).

Self-efficacy of the participant as measured by the Self-Efficacy Scale [SES; (41)]. This 10 items scale has a good internal consistency, Cronbach's α between 0.8 and 0.9 (41).

Coping strategies of the participant as measured by the Brief Coping Orientation to Problems Experienced Inventory (42). The scale consists of 28 items which form 14 subscales, Cronbach's α for all scales range from 0.50–0.90 (42).

#### Secondary Outcomes Parent(s) and/or Legal Guardian(s)

Proxy mental health of the participant at post-inclusion (t3) as measured by the Children Behaviour Check List (CBCL 6-18R; 32). This tool consists of 112 items, the items of the second part can be divided into eight subscales. All eight subscale scores separately and the full scale score will be calculated. For the eight subscales a t-value above 70 and for the full scale above 63 marks the cut-off to a clinically relevant score. Internal consistency of the full scale measured by Cronbach's α is ≥0.93, while it ranges for the subscales from α = 0.59–0.84 in a community sample and from α = 0.62–0.90 in a clinical sample (32).

#### Covariates

The following variables will be included and assessed for their moderating or mediating role in the evaluation of the iCHIMPS intervention.

##### Covariates Participants

Participant characteristics as measured at baseline by self-report items include age, gender, ethnicity, family composition, previous experiences with mental healthcare providers as well as educational and vocational background.

Working alliance between the participant and the E-Coach will be measured by the Working Alliance Inventory for Guided Internet Interventions [WAI-I, (43)]. This questionnaire is based on the Working Alliance Inventory (44) and was adapted for guided online interventions. The 12 items scale can be divided into 2 subscales and has a Cronbach's α of 0.93 for the whole scale, while the two subscales have α's of 0.93 (task and goal agreement) and 0.89 (bond with therapist).

Social support of the participant as measured by the Oslo Social Support Scale 3 [OSSS-3; (45)]. The internal consistency for this short three item scale is 0.64 as measured by Cronbach's α (46).

##### Covariates Parent(s) and/or Legal Guardian(s)

Characteristics of the parent(s) or legal guardian(s) as measured by self-report items including age, gender, ethnicity, family composition, previous experiences with mental healthcare providers as well as educational and vocational background.

Mental health of the mentally ill parent(s) as assessed by the Mini-International Neuropsychiatric Interview [M.I.N.I.; (47)]. This tool was designed to be used by trained interviewers in a face to face setting to assess mental disorders according to DSM-IV and ICD-10 diagnostic criteria. Interrater reliabilities ranging from 0.43 to 0.82 for the different diagnoses have been reported for the interview (48).

Proxy social support of the participant as measured by the Oslo Social Support Scale 3 (OSSS-3; 45). The internal consistency of this short three item scale measured by Cronbach's α is 0.64 (46).

#### Qualitative Outcomes

Open and closed questions concerning the adherence, acceptance, experiences, barriers, facilitators as well as potential drop-outs will be included into the online questionnaire.

#### Economic Outcomes

Health related life quality of the participant assessed by the self-report questionnaire EuroQol-5-Dimensions-Youth [EQ-5D-Y; (49–52)]. The questionnaire consists of two parts. In the first part the participant defines his or her actual health state on the five dimensions mobility, self-care, usual activities, pain/discomfort and anxiety/depression. Each dimension is rated between 1 representing the best health state and 3 representing the worst health state. The overall health state is a 5-digit number representing the rating values for each health state. For the generation of QALYs, the overall health states will be valued using utility value sets generated by means of discrete choice experiments with representative samples from the general population (53, 54). In the absence of a national value set the visual analogue scale provided in the second part of the EQ-5D-Y can be used to value the overall health state.

Utilization of the health care system by the participant as measured by the self-report Children and Adolescent Mental Health Services Receipt Inventory (55, 56). This questionnaire assesses utilization of in- and outpatient health care as well as psychosocial care provided by the child welfare services and school based support provided by the educational system (56).

Health insurance claims data provided by one of the participating German health insurance companies, BARMER, DAK, KKH, TK, MKK, IKK Classic, AOK Baden-Württemberg or AOK Hessen, include information about the utilization of the health care system by members of the participating family who are insured by one of the aforementioned health insurance companies. The claims data consists of all treatment services provided by inpatient and outpatient care, medication, remedies, rehabilitation, and sick leave. All available information can be merged via an pseudonymized identification number for each patient. For every treatment service, all costs from health insurance perspective are documented for the period from January 1^st^ 2018 to June 30^th^ 2022.

#### Negative Effects and Adverse Events

(Serious) adverse events ((S)AEs) will be monitored and assessed in different ways through online questionnaires and structured interviews, adapted from the recommendations of the National Institute for Health Research (57) as well as from Horigian and colleagues (58).

(S)AEs can be reported to the E-Coaches during the intervention or to the study team via email or during the structured clinical interviews.

The deterioration rate will be one measure of possible negative effects of the iCHIMPS intervention. Deterioration is defined as worsening in the assessed mental health of the participant using the Reliable Change Index [RCI; (59, 60)].

Additionally, negative effects of psychological treatments experienced by the participant will be measured during the online assessments by the Negative Effects Questionnaire [NEQ; (61)]. The scale consists of 20 items and is divided into five subscales, namely symptoms, quality of treatment, dependency on the treatment, stigma, and hopelessness. The subscales are ranging in their internal consistency from Cronbach's α 0.72–0.93, while the whole scale shows a Cronbach's α of 0.94 (61, 62).

During the online self-report questionnaires (YSR; items 18 and 93) and during the structured clinical interviews (Kiddie-SADS-PL; depression chapter section 4 and 5) suicidal and self-injurious tendencies and behaviors will be assessed.

#### Completion Times

The Kiddie-SADS-PL interview will take 45 to 75 min to administer and will be carried out at t0 with the participant and the parent(s) as well as at t2 and t3 with the participant.

The M.I.N.I. interview takes ~15 min to administer and will be carried out with the parents with a mental disorder at t0.

Online self-report questionnaires for the participants will take ~60 min at t0, t2 and t3 as well as ~30 min at t1.

Online self-report questionnaires for the participating parents will take ~20 min at t0, t1, t2 and t3.

#### Reimbursement

Only the participating adolescents will be compensated for their participation after the successful completion of the online assessment at t2 and t3 with each 20 €. Reimbursement will be transferred to a bank account of their choice.

#### Data Management, Quality Assurance, and Safety Measures

All interviews will be conducted in person or via phone at the recruiting clinics and at the University Medical Center Hamburg-Eppendorf. The collected pseudonymized datasheets will be stored at the clinics until the end of each quarter and then sent to the study team of Ulm University. At Ulm University, the data will be stored and later transferred to the study team of the University Medical Center Hamburg-Eppendorf to be entered into the data management system provided by the data management company CTC North.

Online assessments will be carried out via the survey platform Lime Survey. The platform is based on servers located at Ulm University. All collected data will be stored pseudonymized. Data from the online assessments will be transferred to CTC North at the end of each quarter.

The CTC North will receive a list of all participants and/or their parents who are insured at one of the participating health insurance companies. Each cooperating health insurance company will then send the health insurance claims data from each participating family member who is a member of their insurance to CTC North.

At CTC North the data from all different sites will be collected and combined. Afterwards the evaluating partners will receive pseudonymized data sets for their respective analyses.

CTC North is additionally responsible for the monitoring of the data management measures at each recruiting clinic.

#### Safety Procedure of (S)AEs

The occurrence of (S)AEs within the diagnostic interviews during the recruitment phase will be handled by the medical health professionals at each clinic, who also decide whether a study participation based on the inclusion and exclusion criteria is possible. Similarly, the occurrence of (S)AEs during the diagnostic interviews at t3, conducted by the study team at the University Medical Center Hamburg-Eppendorf, will be handled by medical health professionals of the local study team.

The occurrence of (S)AEs during the online assessment as well as during the internet-based intervention follows a step wise protocol and clear documentation rules and is handled by the study personnel at Ulm University. In case of (S)AEs, the participant will automatically receive an email with information on help as well as emergency numbers. The study personnel will contact participants who gave critical answers directly. If the participants are not reachable within 48 h on weekday the responsible authorities will be informed.

### Statistical Analysis

#### Clinical Analyses

All statistical analyses will be conducted according to the intention-to-treat principle (ITT). The ITT population includes all study participants of all families that have been randomized. Type 1 error rate (two-sided hypothesis) will be set to 5%. Depending on the level of measurement of each variable descriptive statistics will be reported either for the whole sample or separated by groups.

Due to the fact that family members are subject to common influences, cluster effects are to be expected. Inferential statistics will therefore be conducted with a mixed-models approach (family and participant as random effects). Recruitment site, group membership, time point, and baseline value of each outcome as well as interaction between group membership and time point, as appropriate, will be added as covariates to the model. Endpoints will be operationalized as change from baseline value. Missing values will be direct imputed within the mixed model approach to enable an ITT analysis.

The result of the primary analysis is the between group contrast of the YSR total problems score at t3 (6-months post-inclusion) within the ITT population. Only this comparison will be considered as confirmatory. Additionally, the analysis will be repeated with the per-protocol (PP) population. Secondary endpoints will be evaluated on an exploratory basis with similar models.

#### Economic Evaluation

The cost-effectiveness analysis follows the net benefit method (63–65). To estimate the incremental cost-effectiveness relation (ICER), the incremental costs for additional healthy life years (QALY) through the internet-based intervention will be determined. Cost information is derived from the CAMSHRI-DE assessment tool and additionally from the resource use documented by the claims data. The stochastic uncertainties of the ICER will be estimated by non-parametric bootstrapping (64, 65). Interpretation of the results will be done on the basis of the cost-effectiveness acceptance curve (64, 65). The claims data analysis will be performed in accordance to the Good Practice of Secondary Data Analysis (GPS) guidelines (66).

#### Moderator and Mediator Analyses

Additional moderator and mediator as well as subgroup analyses will be carried out on an exploratory basis with the above defined covariates.

## Discussion

The iCHIMPS IMI, embedded into the multicenter project CHIMPS-NET, offers the opportunity to evaluate the clinical- as well as cost-effectiveness of an innovative form of a guided internet-based self-help intervention for children of parents with a mental disorder. Due to the high digital affinity of the younger generation, this digital mental health promotion offer might resonate especially well with the target audience. Insights from this study might enable us to establish online approaches as a viable mean to support children of parents with a mental disorder, thereby providing much needed mental health promotion offers in a time- and location-independent fashion to this often overlooked population.

The possibility to indirectly compare the iCHIMPS IMI, which is partly based on the CHIMPS-program, to the CHIMPS face-to-face interventions, offers the opportunity to explore the benefits as well as short comings of each individual form of intervention delivery. This could increase the knowledge about when and possibly for whom which mode of delivery can offer advantages over the other, thereby paving the way for a discussion about a more flexible and patient centered approach to prevention and treatment interventions for the children and the whole family.

### Potential Problems and Solutions

Potential problems during the trial might arise from data management, for example failing blinding, problems at data assessment, or storage. To prevent this from happening CHIMPS-NET is supported and supervised by the data management company CTC North. At CTC North the data from different sources will be collected, combined, and then send as pseudonymized data sheets to the evaluating project partners.

To avoid group contamination by participating siblings from the same family CHIMPS-NET has chosen to use a cluster randomized approach.

## Conclusion

The iCHIMPS cRCT will examine the clinical- as well as cost-effectiveness of the internet-based iCHIMPS mental health promotion intervention for children of parents with mental disorders. This provides the opportunity to gain insights into an innovative as well as time- and location-independent form of support for this often overlooked at-risk group. Additionally, the larger CHIMPS-NET project allows indirect comparisons between online and face-to-face interventions for a similar target group.

## Trial Status

Recruitment has started on May 1^st^ 2021.

## Dissemination

Final trial results are planned to be presented on national and international conferences and will be published in peer-reviewed journals. Third party access to the final dataset might be provided depending on the agreement of data security and data exchange regulations.

## Ethical Statement

The present trial was approved by the Ethics Committee of the Ulm University (189/20-FSt/bal.). Additionally, every recruiting adult mental health clinic has obtained an individual approval by their local Ethics Committee in order to be able to recruit participants for this trial. All participating parties, in the case of children below the age of 16, and all legal guardians even if they themselves do not participate in any kind in the study, will have to sign written informed consent prior to any involvement.

## Author Contributions

SW-G, HB, RK, and JZ obtained funding for this study. HB is principle investigator of iCHIMPS. RK and JZ designed the health-economic evaluation and AD the statistical analysis. PD drafted the manuscript. All authors contributed to the final manuscript draft and approved the submitted version.

## Funding

iCHIMPS as part of the larger multicenter study CHIMPS-NET is funded by the Innovation Committee (Innovationsausschuss) of the Joint Federal Committee in Germany (Gemeinsamer Bundesausschuss, GBA no: 01NVF18003).

## Conflict of Interest

The study team of the Ulm University are authors of this manuscript and have developed the internet and mobile-based intervention iCHIMPS. Hence, the final statistical analysis will be conducted by independent evaluators from Ulm University and the Center for Health Economic Research in Hannover (CHERH). HB received consultancy fees, reimbursement of congress attendance and travel costs as well as payments for lectures from Psychotherapy and Psychiatry Associations as well as Psychotherapy Training Institutes in the context of E-Mental-Health topics. He has been the beneficiary of study support (third-party funding) from several public funding organizations. The remaining authors declare that the research was conducted in the absence of any commercial or financial relationships that could be construed as a potential conflict of interest.

## Publisher's Note

All claims expressed in this article are solely those of the authors and do not necessarily represent those of their affiliated organizations, or those of the publisher, the editors and the reviewers. Any product that may be evaluated in this article, or claim that may be made by its manufacturer, is not guaranteed or endorsed by the publisher.

## Abbreviations

AOK: Allgemeine Ortskrankenkasse
APOI: Attitudes toward Psychological Online Interventions Questionnaire
AUC: area under the curve
Brief COPE: Brief Coping Orientation to Problems Experienced Inventory
CAMSHRI-EU: Children and Adolescent Mental Health Services Receipt Inventory
CBCL: Child Behavior Checklist
CBT: cognitive behavioral therapy
CHIMPS-NET: children of mentally ill parents - network
CONSORT: Consolidated Standards of Reporting Trials
CHEERS: Consolidated Health Economic Evaluation Reporting Standards Statement
CHERH: Center for Health Economic Research in Hannover
cRCT: cluster-randomized controlled trial
DAK: Deutsche Angestellten Krankenkasse
DSM: Diagnostic and Statistical Manual of Mental Disorders
EQ-5D-Y: EuroQol-5 Dimensionen-Youth
GBA: Gemeinsamer Bundesausschuss
ICD: International Statistical Classification of Diseases and Related Health Problems
ICC: intra-cluster correlation coefficient
ICER: incremental cost-effectiveness ratio
iCHIMPS: internet-based intervention for children and adolescents of mentally ill parents
IG: intervention group
IKK Classic: Innungskrankenkasse Classic
IMIs: internet- and mobile-based interventions
ISPOR: International Society for Pharmacoeconomics and Outcomes Research
ITT: intention-to-treat
KIDDI-SADS-PL: Kiddie - Schedule for Affective Disorders and Schizophrenia - Present and Lifetime Version
KKH: Kaufmännische Krankenkasse
MDR: Medical Device Regulation
M.I.N.I.: Mini International Neuropsychiatric Interview
MKK: Mobil Krankenkasse
NEQ: Negative Effects Questionnaire
OSSQ: Oslo Social Support Questionnaire
PP: per protocol
QALY: quality adjusted life year
RCI: reliable change index
(S)AE: (serious) adverse event
SAS: Skala zur Allgemeinen Selbstwirksamkeitserwartung
SSRMI: Self-Stigma in Relatives of People with Mental Illness
TAU: treatment as usual
TK: Techniker Krankenkasse
UKE: Universitätsklinikum Hamburg Eppendorf
WAI: Working Alliance Inventory
WAI-I: Working Alliance Inventory for guided internet interventions
WHO: World Health Organization
WHO-5: Wohlbefindens-Index
YSR: Youth Self Report.
